# Reusing sterile cotton fabric barriers in the clinical practice: an observational and longitudinal study ^*^

**DOI:** 10.1590/1518-8345.6645.3990

**Published:** 2023-10-09

**Authors:** Berendina Elsina Bouwman, Dayane de Melo Costa, Francesco Tessarolo, Anaclara Ferreira Veiga Tipple

**Affiliations:** 1 Universidade Federal de Jataí, Jataí, GO, Brasil; 2 Becaria de la Fundação de Amparo à Pesquisa do Estado de Goiás, Brasil.; 3 Universidade Federal de Goiás, Goiânia, GO, Brasil.; 4 University of Trento, Department of Industrial Engineering, Trento, Trentino-Alto Adige, Italy.

**Keywords:** Product Packaging, Textiles, Monitoring, Hospital Equipment Supplies, Sterilization, Asepsis, Embalaje del Producto, Textiles, Vigilancia, Equipos y Suministros de Hospitales, Esterilización, Asepsia, Embalagem de Produtos, Têxteis, Monitoramento, Produtos Médico-Hospitalares, Esterilização, Assepsia

## Abstract

**Objective:**

to analyze the physical and biological barrier characteristics of cotton fields used as a sterile barrier system after multiple use and processing cycles in the clinical practice.

**Method:**

an observational and longitudinal study to monitor and evaluate 100% cotton fabric used as a sterile barrier system in a medium-sized hospital. Samples were collected before use (after three washes) and at three, six, nine, 12 and 15 months of use and evaluated for the number, thickness and integrity of threads, weight, water absorption and wet penetration by microorganisms.

**Results:**

after 85 washes, the number of threads remained unchanged, and the shredded fibers and the water volume absorbed were increased. The microbiological test using the German standard methodology obtained a negative result and wet penetration by microorganisms did not show significant changes over time, although a percentage of the microbial cells passed through the double-layer samples.

**Conclusion:**

the physical properties of 100% cotton used as a sterile barrier system changed with use/processing cycles; however, these alterations did not significantly interfere with the results obtained by the tests performed on the microbiological barrier up to 85 washes.

## Abstract (es)

**Objetivo:**

analizar las características de barrera física y biológica de los campos de algodón utilizados como sistema de barrera estéril después de ser sometidos a múltiples usos y procesos en la práctica clínica.

**Método:**

estudio observacional longitudinal de seguimiento y evaluación de tela 100% algodón, utilizada como sistema de barrera estéril en un hospital de mediano porte. Se recolectaron muestras antes del uso (después de tres lavados) y después de tres, seis, nueve, 12 y 15 meses de uso y se evaluaron el número, grosor e integridad de los hilos, peso, absorción de agua y penetración húmeda de microorganismos.

**Resultados:**

después de 85 lavados, no cambió el número de hilos, aumentó el número de fibras deshilachadas y el volumen de agua absorbida. La prueba microbiológica mediante la metodología estándar alemana obtuvo un resultado negativo y la de penetración húmeda de microorganismos no mostró cambios significativos con el tiempo, a pesar de que una fracción de células microbianas pasó a través de las muestras de doble capa.

**Conclusión:**

las propiedades físicas de la tela 100% algodón utilizada como sistema de barrera estéril cambiaron con el uso/procesamiento; sin embargo, no interfirieron significativamente con los resultados obtenidos en las pruebas realizadas a la barrera microbiológica hasta los 85 lavados.

## Abstract (pt)

**Objetivo:**

analisar as características de barreira física e biológica de campos de algodão, que são utilizados como sistema de barreira estéril, após múltiplos usos e processamentos na prática clínica.

**Método:**

estudo longitudinal observacional de acompanhamento e de avaliação de tecido 100% algodão que foi utilizado como sistema de barreira estéril em um hospital de médio porte. Amostras foram coletadas antes do uso (após três lavagens) e com três, seis, nove, 12 e 15 meses de uso e avaliadas quanto ao número, à espessura e à integridade dos fios, peso, absorção de água e penetração úmida de micro-organismos.

**Resultados:**

após 85 lavagens, o número de fios permaneceu inalterado, porém aumentaram-se as fibras desfiadas e o volume de água absorvido. O teste microbiológico utilizando metodologia padrão alemão obteve resultado negativo; já o de penetração de úmida de micro-organismos não apresentou mudanças significativas ao longo do tempo, embora uma fração das células microbianas tenham passado pelas amostras de dupla camada.

**Conclusão:**

as propriedades físicas do tecido 100% algodão, utilizado como sistema de barreira estéril, alteraram com usos/processamentos; entretanto essas não interferiram significativamente nos resultados obtidos pelos testes realizados na barreira microbiológica até 85 lavagens.

**Highlights:**

**(1)** Clinical use and processing exert an impact on the sterile fabric barrier system.

**(2)** There was weight loss, reduction in size and increase in water absorption volume.

**(3)** The longer the use, the more loose fibers.

**(4)** Penetration by microorganisms did not increase over the 15 months of the study.

**(5)** The physical changes of the fabric did not interfere with the fabric barrier efficiency.

## Introduction

Health Products (HPs) classified as critical and used in invasive procedures must be subjected to sterilization. It is a process capable of destroying all forms of microbial life ^(^ 1 ^)^, whose most commonly used method is Saturated Vapor under Pressure (SVP). To preserve sterility, HPs should be packed in a Sterile Barrier System (SBS) for storage. 

In Brazil, choice of the SBS used for this purpose must be regularized with the National Health Surveillance Agency ( *Agência Nacional de Vigilância Sanitária*, ANVISA), Collegiate Board Resolution ( *Resolução da Diretoria Colegiada*, RDC) No. 15, of March 15 ^th^, 2012 ^(^ 2 ^)^. This resolution provides that the SBS must offer an efficient barrier against microorganisms, particles and fluids in order to allow an aseptic presentation of the HPs and enable proper delivery of the content, preserving them from possible contamination. 

SBS indicated for SVP sterilization are classified into disposable (surgical grade and creped papers and non-woven fabrics) and reusable (cotton fabrics and rigid containers). Cotton fabric has been one of the oldest and most used worldwide until the emergence of non-woven fabrics; its use in Brazil is recommended and remains common in health institutions, although it has not been subject of studies in the last decade ^(^ 2 ^-^ 3 ^)^. 

100% cotton fabric in health institutions is not only used as SBS, but also for making aprons and surgical fields ^(^ 4 ^)^. A recent study that compared the cotton fabric of surgical aprons to disposable fabrics concluded that adopting reusable aprons can result in greater protection and significant cost savings due to their superior durability and sustainability when compared to disposable items ^(^ 5 ^)^. 

To use the fabric, it is recommended to apply it in double layers or in a mixed modality, that is, a layer of fabric and another disposable SBS ^(^ 6 ^)^ and, after each use, it must be washed to remove dirt resulting from storage, transport and use and to restore hydration of the fibers ^(^ 3 ^)^. However, it is understood that the friction to which they are subjected, the cleaning products used and the temperature variations during sterilization, among other elements during processing, may result in variations in their ability to resist penetration by microorganisms according to the number of uses and wash and sterilization cycles ^(^ 7 ^)^. In this sense, some studies have investigated durability and loss of the microbiological barrier of cotton fabric SBS in Brazil; however, their findings are divergent regarding the ideal number of processing cycles (washing and sterilization) safe for use ^(^ 8 ^-^ 9 ^)^. In addition to that, there are difficulties in reliably recording the processing frequency of each surgical textile ^(^ 10 ^-^ 11 ^)^. 

Considering the lack of consensus regarding the safe number of use and processing cycles and the challenges to evaluate maintenance of the cotton fabric properties for health institutions and professionals, especially nurses (historically the technical professionals in charge of the Central Sterile Supply Department [CSSD] unit), the following guiding question was established for this study, a pioneer in the evaluation of the properties of cotton fabrics used in the clinical practice: Are there changes in the physical characteristics and biological barrier of 100% cotton fabric fields used as sterile barrier systems subjected to multiple use and processing cycles in relation to new ones? And, as objective: to analyze the physical and biological barrier characteristics of cotton fields used as sterile barrier systems after multiple use and processing cycles in the clinical practice.

## Method

### Study design

A longitudinal and observational study to monitor and evaluate SBS samples made with 100% cotton fabric and used in the clinical practice, during a 15-month period. The Strengthening the Reporting of OBservational Studies in Epidemiology (STROBE) guide was used to report the research.

### Context

The study was carried out between February 2018 and May 2020 in a medium-sized philanthropic hospital (n=60 beds) from Jataí, Southwest region of the state of Goiás (GO), Brazil. Samples of 100% single and double cotton fabric from fields used as SBS, new and after subsequent processing cycles, were analyzed. The samples were analyzed in the Bacteriology and Mycology Laboratory of the Biomedicine Course at the Federal University of Jataí and in the Department of Medicine Laboratory, Provincial Health Services Trust of Trento/Italy - Santa Chiara Hospital – Trento, Italy.

### Definition of the samples

Based on the demands of the institution’s surgeries (mean of 101 monthly surgeries), 104 100% new cotton fabric fields [1.20 meters (m) x 1.20 m] were made, plus 30% for replacements during the research (31 fields). The fabric used met the standard of the Brazilian Association of Technical Standards ( *Associação Brasileira de Normas Técnicas*, ABNT) - Brazilian Standard ( *Norma Brasileira*, NBR) 14,027/9 ^(^ 12 ^)^, of the Santista ^®^ brand and classified by Solasol in royal blue color (518/193952TC D), 100% cotton, texture of approximately 39.63 threads *per* square centimeter (cm ^2^), weight of 260 grams *per* square meter (g/m ^2^) and 3/1 twill ligament. Although this standard indicates 2/1 ligaments, a 3/1 presentation was used to achieve the recommended weight. 

In each piece, a chart containing 120 spaces was printed in order to monitor the number of washes to which it would be subjected. Thus, when receiving the laundry fields, in the CSSD area devoted to folding, the nursing technician filled a space with black indelible pen ^(^ 13 ^)^. The stage was monitored daily by a researcher and/or research assistants (two), previously trained, during the 15-month study period. Before the first use, the new fields were washed three times to remove the starch. 

### Data collection

Samples for the analyses were collected at the CSSD folding area when the textiles were received from the laundry, selecting three different fields (one sample was cut from each of the three fields) at each collection, selecting those that contained the record of the largest number of washes. The collection moments were as follows: “Zero” time: after the third wash and before the first use; Time 1: with three months of use; Time 2: with six months of use; Time 3: with nine months of use; Time 4: with 12 months of use and Time 5: with 15 months of use.

All new fields were washed three times before the first use to remove the starch used in the ironing process by the manufacturer. Three fields were randomly selected at this stage as samples for the Control Group (not subjected to use/processing cycles).

### Analysis and statistics

**Figure 1 -:**
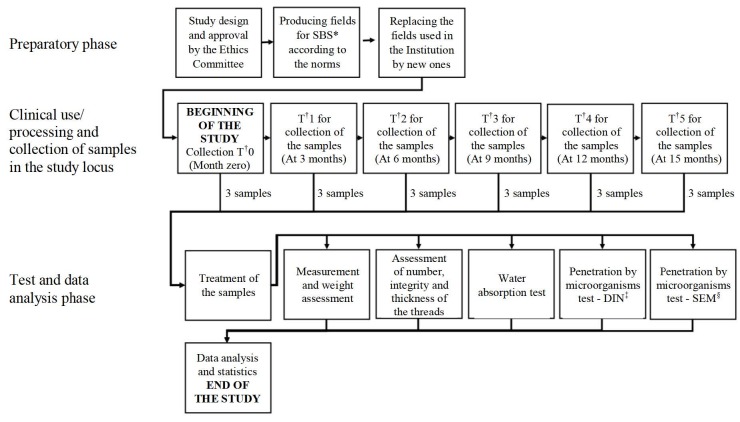
Sequential distribution of the study design containing the preparatory phase, collection period of cotton fabric samples and number, as well as analytical tests performed and data analysis. Jataí, GO, Brazil, 2018-2020

Figure 1 represents the study design from the preparatory phase, collection period and number of samples and analytical tests performed to data analysis. Figure 2 shows the analytical tests performed, the place where they were applied, and the number, size, shape and fabric layers of the samples. 

*Sterile barrier system; †Time; ^‡^German Standard Methodology; ^§^Scanning Electron Microscopy

**Figure 2 -:** Analytical tests performed, place where they were applied, and size, shape and number of both the fabric layers and the samples used. Jataí, GO, Brazil, 2018-2020

Tests	Sample size and shape	Fabric layers	Number of field samples					
			Months of use					
			0	3	6	9	12	15
Measurement and weight assessment *	Square (15 cm ^2^)	Simple	3	3	3	3	3	3
Assessment of the number of threads *	Circle (12 mm diameter)	Simple	1	1	1	1	1	1
Assessment of the integrity and thickness of the threads †	Circle (12 mm diameter)	Simple	1	1	1	1	1	1
Water absorption test †	Rectangle (1.5 cm x 8 cm)	Simple	3	3	3	3	3	3
Penetration by microorganisms suspension test - German Standard Methodology 58.953 *	Square (10 cm x 10 cm)	Double	3	3	3	3	3	3
Penetration by microorganisms suspension test – Scanning Electron Microscopy - SEM †	Circle (24 mm diameter)	Double	3	3	3	3	3	3

*Bacteriology and Mycology Laboratory of the Biomedicine Course at the Federal University of Jataí ( *Universidade Federal de Jataí*, UFJ); †Department of Medicine Laboratory, Provincial Health Services Company of Trento/Italy - Santa Chiara Hospital – Trento, Italy

Measurement of the fields was verified at the determined times, using a ruler with 1 millimeter (mm) precision. The mean of the three fields was calculated in m ^2^, for each period analyzed. The weight of the samples *per* unit area of fabric (g/m ^2^) was obtained by weighing a 15×15 cm ^2^ specimen on an electronic scale (Edutec ^®^/Brazil), with 0.001 g resolution, also through the mean value obtained by the three samples. The number of threads in the weft and warp was counted with a magnifying glass (Foldable Magnifier ^®^/Brazil). 

Through Scanning Electron Microscopy (SEM) (Fei ^®^ Quanta 200 F- United States), thickness and integrity of the threads (warp and weft direction) and the changes at the different use times were analyzed. From each sample, three front and back images were taken with 100× and 250× magnification, as well as one image at 500× [5 kilovolt (kV) voltage and 0.45 Torr pressure]. The Image J program (National Institutes of Health) was used to measure thickness of the threads. Six measurements were performed in different locations of the anterior and posterior parts of the sample, calculating the mean obtained in each sample and, subsequently, the mean obtained from the three samples with the same use time. 

For the water absorption evaluation, the mean of the three weights obtained from the samples (strips of 8 cm in length and 1.5 cm in width) with the same use time was considered. A Wilhelmy Plate Method microscale (DCA 322 CahnTM ^®^, Netherlands) and high purity water [0.2 micro-Siemens *per* centimeter (µS/cm)] were used. The fabric strip was hung on the microscale hook and the free tip was brought into contact with water. Weight gain with the water absorption time was recorded. The modified Washburn equation was used to adjust the linear dependence of the squared water mass *versus* time. This approach allowed calculating a linear coefficient, which described the water absorption characteristics of the samples that can be affected by use and processing due to changes in fabric absorption and capillary structure. 

Two methodologies were used in the microbial barrier analysis, namely: German Standard Methodology ( *Deutsches Institut für Normung*-DIN) 58,953 - Part six (2016), to semi-quantitatively evaluate microbial penetration through the cotton fabric samples (both tested in single and double layers), and a new SEM-based test protocol developed for this study. 

For the DIN methodology, 10 cm ^2^ fabric samples were marked with five circles after sterilization, using an indelible pen. Aseptically, in a chapel with laminar flow on sterile Petri dishes and with the aid of an automatic pipette, 100 microliters (µl) of *Staphylococcus aureus* (ATCC ^®^25923) suspension were applied to 10 milliliters (mL) of 0.9% sterile saline solution (sodium chloride), obtained from the 1/100 dilution of the McFarland 0.5 scale, in each circle marked in the sample. Subsequently, the samples remained in the chapel with laminar flow under ventilation system for six hours. 

At the end of this period, the back of the fabric samples was pressed for five seconds with the aid of another sterile medium-sized Petri dish on the surface of a plate containing nutrient agar. Subsequently, the culture medium was incubated at 35 degrees Celsius (°C) for 72 hours. The Colony Forming Units (CFUs) were counted on the plates every 24 hours, calculating the mean of the three samples. As established by the methodology, the test result is considered positive when more than 5 CFUs are detected in a sample.

The wet microbial penetration test using Scanning Electron Microscopy consisted of adding 100 µl of *S. aureus* (ATCC ^®^6538) suspension containing 6 mL of sterile distilled water (0.55 absorbance) to the circular 24 mm samples of double fabric, with the aid of an electronic pipette. After 30 minutes, it was examined by means of SEM (5 kV voltage, 6,000x magnification and 0.68 Torr pressure) to check for bacteria penetration through the fabric on the back of the second layer of the samples at random locations. The mean number of coccus cells seen in the view field (2,130 square micrometers-µm ^2^) was calculated in all three samples of each study period. 

The statistical analyses were performed using the Prism 5 statistical software (GraphPad Software, San Diego, CA, USA). The categorical variables were expressed in percentages. Median and Interquartile Range (IQR) were used in the results of the continuous variables. The median values obtained from the different periods were qualitatively compared to evaluate potential trends (increasing, decreasing or unchanged) corresponding to the variables of interest.

Significance of the differences between each test group and the Control Group was analyzed with Mann-Whitney’s U test for the continuous variables and with Fisher’s Exact test for the dichotomous variables. Bonferroni-Holms *post-hoc* correction was considered for multiple comparisons. Bilateral tests were considered and the statistical significance level was set at p<0.05. 

### Ethical aspects

The study was approved by the Ethics Committee of the Federal University of Goiás (Certificate of Presentation for Ethical Appraisal Protocol No. 64541517.3.0000.5083) and had the consent of the hospital management and the technicians responsible for the CSSD and the Surgical Center ^(^ 14 ^)^. 

## Results

The mean washings of the cotton fields used with SBS in each study period were as follows: 24 times - three months; 42 times - six months; 57 times - nine months; - 70 times - 12 months and 85 times - 15 months.

Table 1 presents a comparative synthesis of the results obtained from the analytical tests for the characterization of 100% cotton fabric fields employed as sterile cotton barrier systems, unused and used in the clinical practice and processed. 

**Table 1 -:** Comparative synthesis of the results obtained from analytical tests for the characterization of 100% cotton fabric fields employed as sterile barrier systems, unused (Control Group) and used in different use/processing time periods in the clinical practice. Jataí, GO, Brazil, 2018-2020

Variable of interest	Unused samples*	Samples used and processed* † *% changes in relation to the unused samples*					Variable trend ^‡^
		Sample use time (months)					
			6	9	12	15	
**Measurement (m** ^2^ **)**	1.44 (1.44-1.44)	**1.33 (1.32-1.33)** -7.6% P<0.001	**1.32 (1.32-1.32)** -8.3% P<0.001	**1.30 (1.29-1.32)** -9.7% P<0.001	**1.31 (1.30-1.32)** -9.0% P<0.001	**1.29 (1.29-1.30)** -10.4% P<0.001	↓
**Weight (g/m** ^2^ **)**	284 (283-285)	287 (285-288) *+1.1%*	284 (281-286) *0.0%*	**276 (274-276)** -2.7% P=0.035	274 (271-276) *-3.4%*	**267 (267-269)** -5.9% P=0.003	↓
Number of threads	40 (40-40)	40 (40-40) *0%*	40 (40-40) *0%*	40 (40-40) *0%*	40 (40-40) *0%*	40 (40-40) *0%*	↔
Measurement of the thread in the weft (µm)	1003 (984-1044)	816 (808-834) -18,6% P=0.003	726 (708-766) -27,6% P<0.001	763 (749-769) -23,9% P<0.001	704 (693-715) -29,8% P<0.001	734 (726-764) -26,8% P<0.001	↓
Measurement of the thread in the warp (µm)	1937 (1934-1942)	*2009 (1934-2015)* *+3.7%*	*1925 (1881-1946)* *-0.7%*	*1919 (1881-1946)* *-1.0%*	1797 (1785-1836) -7.2% P=0.005	1754 (1732-1770) -9.5% P<0.001	↓
**Water absorption test (mg** ^2^ **/s)**	289 (287-290)	2579 (2448-2627) +792% P<0.001	2778(2719-2784) +861% P<0.001	2755 (2685-2797) +853% P<0.001	2617 (2532-2629) +806% P<0.001	2435 (2059-2600) +743% P=0.016	↑
**Penetration by microorganisms suspension test - German Standard Methodology 58.953 (Number of positive samples)**	0 (0-0) 0%	*0 (0-0)* *0%*	*0 (0-0)* *0%*	*0 (0-0)* *0%*	*0 (0-0)* *0%*	*0 (0-0)* *0%*	↔
**Penetration by microorganisms suspension test – Scanning Electron Microscopy (Bacterial cells/6000x)** ^§^	13 (9-17)	*18 (11-23)* *+39%*	*17 (9-18)* *-31%*	*10(9-20)* *-23%*	*8 (7-14)* *-39%*	*13 (12-15)* *0%*	↔

*Values expressed as median (first quartile-third quartile) of the distribution corresponding to the experimental values; †Values are reported in bold when significantly different from the Control Group, p<0.05; ^‡^Qualitative comparison corresponding to the values of the variables over the entire life cycle of the device: ↑ increase; ↔ unchanged; ↓ decrease; ^§^The 6000× view field corresponded to an area of 0.00213 mm ^2^

The weight assessment related to size (g/m ^2^) of the fields before and in subsequent use periods, in months, showed that there was progressive dimensional shrinkage in all fields and, in parallel, an increase in weight up to three months of use, of 1.1% (g/m ^2^), when compared to those unused. After this period, a gradual 5.9% (g/m ^2^) reduction was verified up to 15 months of use ( Table 1). 

**Figure 3 -:**
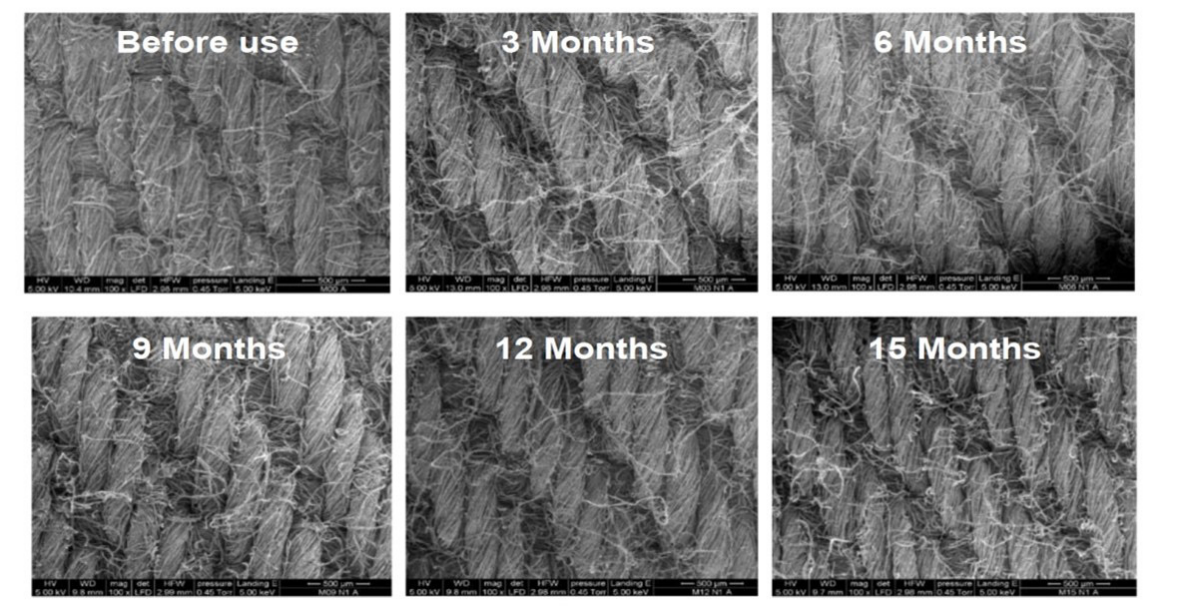
Images obtained by means of Scanning Electron Microscopy of samples of 100% cotton fabric fields intended for sterile barrier systems before and in subsequent use periods, in months [three (Mean - M): 24 washes), six (M: 42 washes), nine (M: 57 washes), 12 (M: 70 washes), 15 (M: 85 washes)] at a medium-sized general hospital. Jataí, GO, Brazil, 2018-2020

The analysis performed to verify the number of threads in the warp and in the weft of the samples presented no changes, remaining at 40 threads *per*cm ^2^. However, the longer the use time, the higher the number of fibers of the fabric threads shredded, as can be seen in the images of the samples obtained by means of SEM, shown in Figure 3. 

Thickness of the threads in the weft of the three-month samples presented a 187 µm (-18.6%) decrease in relation to the samples before the first use (three washes). At 12 months (M: 70 washes), a 299 µm (-29.8%) reduction was observed; this result was statistically significant ( Table 1). 

*Department of Medicine Laboratory, Provincial Health Services Company of Trento/Italy - Santa Chiara Hospital – Trento, Italy

There was a statistically significant difference in the water absorption volume between the samples before the first use and those with three, six, nine, 12 and 15 months of use ( Table 1). However, the difference between the water absorption volumes in the time intervals after use was not statistically significant. 

The results of the microbial tests according to the DIN methodology showed that the percentage of cells that passed through the cotton fabric field was 0.002% (2 CFUs/10 ^5^cells) at the most, considered negative (<5 CFUs), which means that all samples met the test requirements both for single and double fabric samples, as shown in Table 1. 

The penetration by microorganisms analysis by means of SEM of double fabric samples ( Table 1) revealed that there was transfer of bacteria in the tested samples from all periods analyzed in the study. The mean number of bacteria seen increased proportionally to the use time until the sixth month (mean of 42 washes), when it presented the highest penetration number, with a mean of 17 bacteria. The samples at nine months (mean of 70 washes) and 12 months of use (mean of 85 washes) presented lower mean bacteria values: ten and eight. These results did not present any statistically significant difference. 

## Discussion

The analysis of the fields used as SBS, in clinical use and control of the number of use and processing cycles, evidenced that there was an increase in weight (g/m ^2^) up to three months of use, despite dimensional shrinkage, when compared to the unused fields. In addition, after this period, a gradual loss was verified up to 15 months of use. These results are similar to those of an experimental study that followed the phases of washing and sterilization of fields for double cotton SBS and where the samples (100 cm ^2^) showed increased weight (g/m ^2^) up to the 25 ^th^wash and, subsequently, a gradual decrease up to the 75 ^th(^ 9 ^)^; they also match another research study ^(^ 4)^), carried out with 100% cotton fabric samples from surgical fields and aprons with the same characteristics of this study and subjected to multiple use and processing cycles in the clinical practice. 

Another study was carried out in a Basic Health Unit (BHU) with cotton fabric fields used in the Unit as SBS, from three manufacturers; the fabric was processed in commercial washers and dryers and sterilized in a saturated steam autoclave under 21 liter pressure. Samples (4 cm ^2^) subjected to 65 processing cycles were evaluated, which evidenced that the samples from two manufacturers (2x1 ligaments/weft) presented a reduction in weight (g/m ^2^), whereas those from one manufacturer (3x1 ligaments/weft) showed an increase ^(^ 15 ^)^. 

Regarding the weight loss of the textiles, another study ^(^ 9 ^)^, in which the fields were processed in a hospital washer, centrifuge and dryer by a third-party company and not used in the clinical practice, the mean loss that began after the 25 ^th^wash was 5.1% after 65 washes. In another study ^(^ 15 ^)^, where the fabrics were processed in a commercial washer and dryer and used as SBS in a BHU, the mean weight loss of the fields from two manufacturers was 0.1% and 0.5% after 65 washes, respectively. Values lower than the one found in this paper (mean of 8.4%), whose fields used with SBS underwent processing in a washer, centrifuge and dryer (15 months of use, mean of 85 washes) showed that there was greater wear out of the fabrics used in the clinical practice at the hospital. 

Despite the mean 8.4% reduction in the weight of the fields in this research, the fabric presented compliance regarding the minimum weight established by NBR 14,027 (simple fields), weighing more than 210 g/m ^2^( ^( 12)^ )^. It is noted that this property was tested exclusively in the fabric evaluated in this study, not allowing to infer the same for other textiles, although it is presumable for those that follow the same specifications. 

The longer the use time and number of washes of the fields used as SBS, the higher the number of shredded and loose fibers. Despite the damages to the structure, it was evidenced that there was no change in the number of threads, both in the warp and in the weft, up to 15 months of use and 85 washes. Similar results were reported in a study conducted with cotton fabric masks ^(^ 16 ^)^. 

The aforementioned study ^(^ 15 ^)^, carried out in a BHU, also analyzed the number of shredded and loose fibers, presented similar results and evidenced maintenance of the number and a reduction in the thickness of the threads and an increase in detachment of fibers from double cotton fields from three manufacturers, after 65 processing cycles (washing and sterilization). It also verified that there was a difference in degradation performance between the evaluated samples from the same manufacturer and from one manufacturer to the other. 

It is also worth noting that, despite the physical changes identified by the analytical tests, all samples were visually intact and none had mending, which are minimally expected conditions for the useful life of a 100% cotton fabric SBS. These facts reinforce the importance of these indicators in defining the finitude of this type of SBS in the care practice.

Water absorption was higher in the samples that had three months (24 washes) when compared to those before the first use (three washes). Considering that SVP sterilization, a method to which HPs packed with cotton SBS are subjected, this result favors the prerogative of a barrier system that should allow penetration and removal of the sterilizing agent during the sterilization process ^(^ 2 ^-^ 3 ^)^. However, it is recommended that, at the end of the sterilization cycle, the packages be dry and stored in clean and dry places, handled as little as necessary, and that the storage conditions are periodically inspected in order to maintain sterility ^(^ 1 ^,^ 17 ^)^. Therefore, this result reinforces the disadvantage of cotton fabric’s low repellency to liquids ^(^ 3 ^)^, an aspect that deserves to be further investigated. 

Based on the prerogative that the fabric fields used as SBS of HPs should guarantee sterility of these products during the storage period, in the 1990s, in the Manual of Processing for Articles and Surfaces in Health Services ^(^ 18 ^)^, the Ministry of Health recommended a shelf life of seven days for the storage of HPs packed in cotton fabrics, which could be extended by validating the storage conditions of each service. In this sense, studies that aimed at analyzing how sterility of HPs is maintained and, therefore, the barrier offered by cotton fabric as SBS, obtained results that extended the period recommended in that manual. 

A survey ^(^ 19 ^)^ that evaluated SPV-sterilized HPs packed in cotton fabric concluded that, after 25 days of storage under controlled conditions, there was no microbial growth in the HPs. Another two studies ^(^ 20 ^-^ 21 ^)^ evidenced that the 100% double cotton fabric maintained sterility of the HPs for periods of 148 and 18 days, respectively. It is noted that the most current recommendations regarding the storage time for HPs subjected to sterilization consider that the period must be based on the SBS custody and integrity conditions ^(^ 1 ^,^ 3 ^)^. 

The result of the penetration by microorganisms microbiological test using the DIN 58.953 methodology - Part six ^(^ 22 ^)^, with the samples of single and double SBS fields, presented a negative result; therefore, the microbiological barrier was maintained until the end of the study (85 ^th^ wash). This number of washes was higher than the one found in the experimental study ^(^ 9 ^)^ carried out at the laboratory, where the microbial barrier of double cotton fields intended for SBS was evaluated, with different numbers of processing cycles and using the same methodology (DIN) and inoculum of the test applied in this study. With double fabric samples, the authors identified that, after the 70 ^th^wash, there was penetration by microorganisms in four fields: 22, 30, 66 and 55 CFUs. Based on these results, the authors recommended 65 as the limit number of washes in fabrics used for SBS. 

The double cotton fields evaluated in the aforementioned paper ^(^ 9 ^)^ were not used in the clinical practice, as were the ones assessed in this study. Due to greater wear out in the fields used in this study as a result of use/processing cycles, more bacterial cells penetrating through the samples were expected. Thus, an increase in the number of shredded and loose fibers of the sample threads was also observed in this study, with some sort of “web” forming in the pores that may have hindered passage of the bacteria in fields with longer use times. 

Considering the findings of the studies that evaluated microbial growth in SVP-sterilized HPs ^(^ 19 ^-^ 21 ^)^ and the results of the DIN methodology test used in this study and in the aforementioned one ^(^ 9 ^)^, it can be asserted that the fabric offers an effective microbiological barrier, although without consensus on the maximum frequency of washes to guarantee the barrier. 

These results differ from the other test performed in this research, which had the same purpose of evaluating the microbiological barrier, but through SEM. The images obtained showed presence of *S. aureus* on the back of all samples tested. Therefore, it is assumed that the use and processing times did not interfere with penetration by microorganisms, as it was the case since the first evaluation (three washes). 

If, on the one hand, the methodology adopted by SEM presents the limitation of using microorganisms in liquid media, easing transfer, which is considered the worst possible scenario, it is known that humidification of an SBS occurs exclusively during the sterilization cycle ^(^ 3 ^)^ and that the HP must be kept dry after packaging; on the other hand, it is necessary to emphasize that the DIN 58.953 - Part six ^(^ 22 ^)^ methodology was developed to evaluate the barrier made of papers used as SBS, which may not be effective to evaluate the barrier of reusable fabric fields used as SBS. 

A research study ^(^ 23 ^)^ reported that penetration of bacteria ( *Geobacillus stearothermophilus* and *Bacillus atrophaeus*) applied dry in three different fabric samples tested (100% tencel [fabric made of cellulose, with a silky texture]; 50% cotton/50% polyester; fabric made with two layers of polyester and one of polyurethane), showed a reduction in microorganism permeability, with the lowest index found in the 50 ^th^processing cycle when compared to samples with only one wash and sterilization cycle. The results are similar to those found in this study, despite the differences related to the fabric, type and use of dry microorganisms. 

The same authors ^(^ 23 ^)^ also evaluated maintenance of the sterility of HPs packed with the same three types of fabric after one, 10, 20, 30 and 50 washes. After sterilization, the HPs were stored for up to three months on shelves in an environment with temperature control between 15°C and 30°C and 30% to 60% relative air humidity. It was concluded that the fields submitted to 50 washing cycles, used with SBS, presented microbial barrier properties within three months of storage, as there was no microbial growth in the evaluation of the products packed in these fields. Therefore, the results evidenced that fabric samples from all periods tested and that had passage of microorganisms in the dry penetration test showed efficiency in maintaining sterility of the HPs packed in them for up to three months under controlled conditions. Such results are in line with the aforementioned studies that evaluated how sterility was maintained ^(^ 19 ^-^ 20 ^)^. 

Preservation of HP sterility was also analyzed in a study ^(^ 24 ^)^ aimed at determining if there is contamination of sterile products when stored under high temperature and humidity. The results showed that there was no microbial growth. Thus, in adverse conditions, the internal content of the boxes packed with cotton fabric fields maintained sterility, proving to be an effective barrier. However, it should be noted that, in case of an adverse event where the sterile material with cotton fabric SBS comes into contact with liquids, there may be contamination and it should be disregarded due to the capacity of liquid absorption and passage of microorganisms in wet conditions, as shown in the results of this study. 

It is worth noting that similar results in physical properties and performance of the microbial barrier were obtained by testing surgical fields and aprons for 15 months applying the same test methodology ^(^ 4 ^)^. 

This study has a limitation related to the number of samples available in each period, although enabling a broad approach with multiple analytical tests. Another limitation was the use of originally developed tests instead of standardized methodologies. This approach allowed for a more precise quantitative comparison between the test and control groups, whereas standard tests are better suited to verify compliance with prerequisites, oftentimes using a qualitative approach.

The results of this study cannot be extended directly to other fabric compositions and further studies are required to evaluate the effect of alternative methods and technologies for processing reusable fabrics.

In this sense, by contributing to defining minimum indicators for the use and reuse of SBS made of 100% cotton fabric and their sizing in health services, this study also contributes to the work process of Nursing teams, which are the main players in CSSDs, especially in Brazil. Additionally, it is relevant for the nurses who make up the Committee on Processing of Health Products and the Commission on Healthcare-Associated Infections.

## Conclusion

It is concluded that fields intended for SBS, made of 100% cotton fabric, did not change the number of threads of the samples after use and the repeated washing and sterilization processes; however, there was impairment of the fabric’s physical structure, shown by the increase in the number of shredded fibers of the threads and in the mean volume of absorbed water.

While a significant change in the fabric’s physical properties has been documented over the 15 months of clinical use and processing cycles, the microbiological tests showed that no significant degradation of the microbial barrier properties was observed over time.
